# Picosecond Yb-doped tapered fiber laser system with 1.26 MW peak power and 200 W average output power

**DOI:** 10.1038/s41598-020-74895-z

**Published:** 2020-10-20

**Authors:** Andrey Petrov, Maxim Odnoblyudov, Regina Gumenyuk, Lidiya Minyonok, Andrey Chumachenko, Valery Filippov

**Affiliations:** 1 Peter the Great St. Petersburg Polytechnical University, Polytechnicheskaya ul. 29, St. Petersburg, 195251 Russia; 2 Tampere University, Korkeakoulunkatu 3, 33720 Tampere, Finland; 3 Ampliconyx Ltd., Lautakatonkatu 18, 33580 Tampere, Finland; 4 Saint-Petersburg National Research University of Information Technologies, Mechanics and Optics, Kronverkskiy pr. 49, St. Petersburg, 197101 Russia

**Keywords:** Fibre lasers, Mode-locked lasers, Ultrafast lasers

## Abstract

We demonstrate a compact picosecond master-oscillator power-amplifier (MOPA) system based on an Yb-doped polarization-maintaining double-clad tapered fiber (T-DCF) delivering pulses with over 1.26 MW peak power and average output power up to 200 W preserving near diffraction limited beam quality. The unique properties of an active tapered fiber enable to amplify the seed pulses directly with no need for applying of additional stretching technique. This simplified laser system can find the practical implementation in industrial micromachining.

## Introduction

The technology of picosecond lasers for industrial and medical applications has been progressing rapidly over the last decade^1,2^. Due to their unsurpassed quality, picosecond lasers can reach a new level of microprocessing accuracy in various industrial applications—microelectronics, semiconductor and photoelectric industry. The energy accumulated in a short laser pulse allows obtaining high peak powers creating unique conditions for studying ultrafast processes and nonlinear effects, as well as for processing and modifying the properties of materials. Such short pulses entitle to avoid undesirable thermal effects and enable the processing of various materials that are almost impossible to process using continuous wave (CW) lasers such as glass, ceramics, carbon fiber reinforced plastics, high temperature alloys, transparent materials.

Currently, most of the high-power picosecond laser systems used in industrial applications are based on bulk solid-state lasers. However, such systems are relatively expensive and large, and they require regular maintenance. Moreover, commercially available bulk solid-state picosecond lasers have limited average output power, typically up to 100 W, and capable to support only low repetition rates, typically up to 1 MHz, while maintaining sufficient pulse energy of about 100 µJ. This limits application of bulk solid-state lasers in the fields where high processing speed over the large area parts is required. In contrast, fiber lasers offer functionality, simplicity, durability, high efficiency, compactness, and a relatively modest price^3^. Therefore, there is strong industrial demand for high-power picosecond fiber lasers with output performance comparable to bulk solid-state lasers, which enable to operate in a wide range of repetition rates.

However, the performance of direct amplification of picosecond fiber lasers is still limited due to strong nonlinear effects^4^. They occur in insufficiently large mode area of active fibers maintaining single-mode operation even at microjoule level of picosecond pulse energy. This fact restrains the achievable peak power below megawatt level essential to implement high quality ablation process in a wide range of materials.

The most common approach to boost peak power and pulse energy when using fiber amplifiers is chirped pulse amplification (CPA)^5^. The CPA technique encloses stretching of the seed pulse up to several hundred picoseconds or even several nanoseconds scale before the last stage of amplification to reduce peak power level inside the fiber amplifier substantially below Stimulated Raman Scattering (SRS)^6^ threshold. The CPA system is completed by subsequent pulse compression down to several picoseconds or even to hundreds of femtosecond scale using bulk dispersive elements. This approach enables to obtain from several tens up to hundreds MW of peak power. However, CPA technology leads to a complication of laser design and significant increase in the cost of the system and its size, which makes it reasonable only for implementation in femtosecond fiber laser systems^7^. Therefore, there is a strong practical demand to develop cost effective simple-to-use fiber amplifier technology to build a compact picosecond laser system capable to deliver MW scale peak power, few tens of μJ of pulse energy and over 100 W of power handling in result of direct amplification of low power signal without involvement of CPA technique. An additional benefit of such high power laser system would be a possibility to get pulse energy sufficient for material processing at high repetition rate to fabricate large area parts with high speed and precision.

A compact laser system with direct amplification of picosecond pulses can be realized using active tapered double-clad fibers (T-DCF)^8,9^. The T-DCF is a fiber with longitudinal geometrical profile allowed support of fundamental mode propagation from a narrow part to a wide part of the fiber. The T-DCF fiber has several advantages: (1) increased pump absorption due to the efficient mode mixing, (2) possibility to use cost-effective, low brightness^10^ and high power multimode source as a pump^9^. The fundamental properties of T-DCF were extensively studied experimentally and theoretically in the past, and details can be found elsewhere^11–13^.

In this work, we demonstrate a possibility to achieve 200 W average power and 1.26 MW peak power from a compact picosecond MOPA (master-oscillator power-amplifier) system with near diffraction limited beam quality (M^2^ ~ 1.34). The laser system comprises a 50 μm core polarization-maintaining T-DCF-based amplifier seeded by 40 ps mode-locked fiber laser operating at wavelength of 1.04 μm with the modest output power of 10 mW. We have also designed and manufactured a compact fiber-coupled micro-optics-based pump unit (multiplexer) capable to withstand up to 300 W of the pump power to complete the system functionality. These results open the door to commercial application of a T-DCF for manufacturing of a high-power all fiber picosecond laser system for industrial precision machining.

## Materials and methods

### High power double-clad tapered amplifier

A high-power picosecond amplifier was composed of an Yb-doped double-clad polarization-maintaining tapered fiber commercially available from Ampliconyx Oy. The measured in-core and clad absorptions were 900 dB/m and ~ 25 dB/m at 976 nm, correspondingly. The numerical aperture (NA) of the core was 0.09, and the first clad NA was 0.28. An ytterbium double-clad tapered fiber had cladding-to-core diameter ratio (CCDR) of 10 with output core diameter of 50 μm. The input core diameter was matched to the output fiber of the seed source (10 μm) to minimize the splicing loss. The total length of the T-DCF was about 2.5 m.

**Figure 1:**
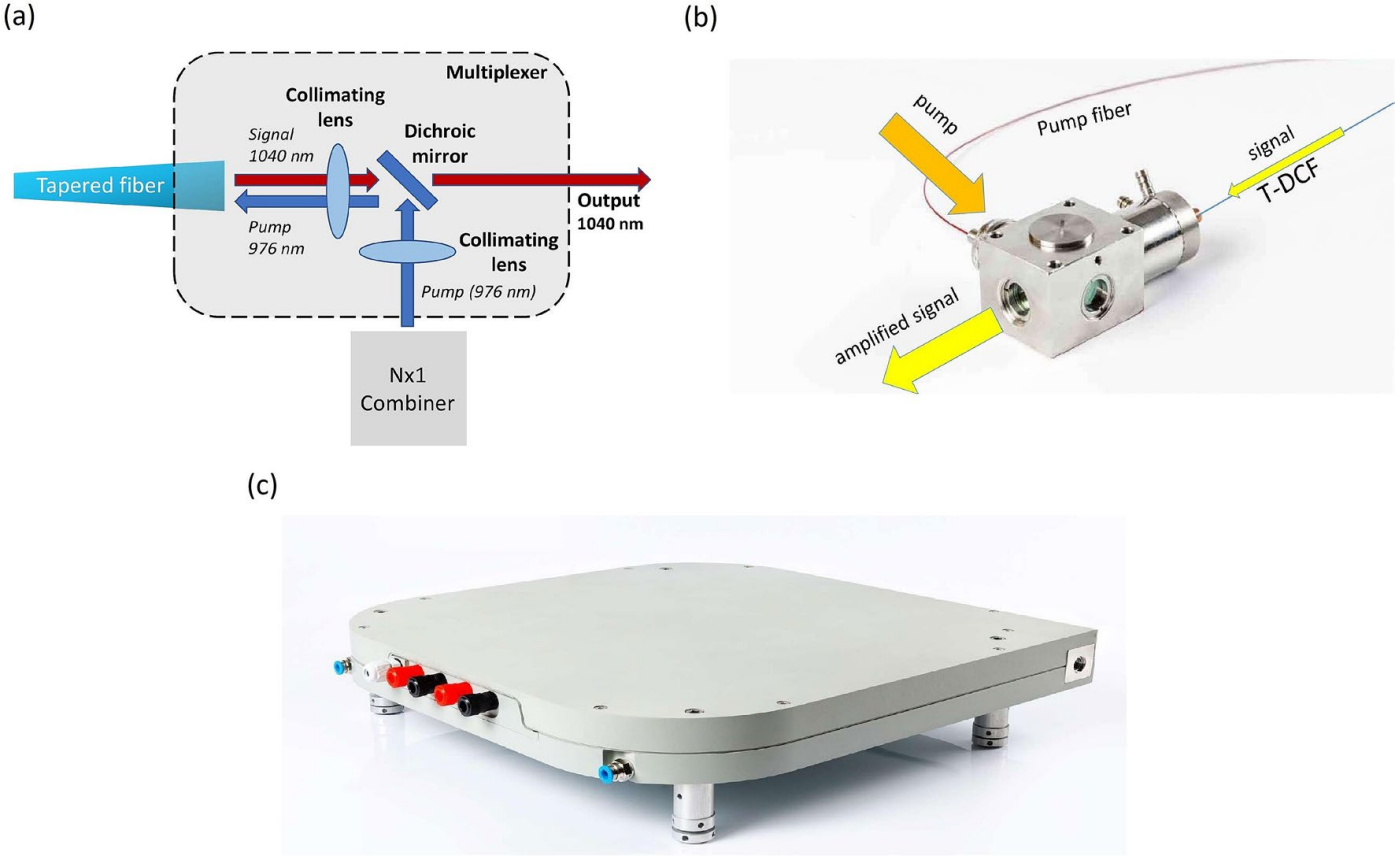
(**a**) Schematic of the multiplexer. (**b**) A micro-optic-based multiplexer. (**c**) A packaged fiber amplifier based on T-DCF.

The T-DCF amplifier was designed to operate in a counter-propagating scheme shown in Fig. 1a. The beam from 976 nm multimode pump source was collimated and directed at 90° via the dichroic filter, and then focused by the second lens at the T-DCF end face. The filter was transparent for outgoing amplified signal at 1040 nm. The pump source included three wavelength-stabilized laser diodes delivered 100 W each via 105/125 μm output fiber. The pump laser diodes were spliced to N × 1 combiner with 220/240 μm output fiber. We assembled the multiplexer in a compact water-cooled package with the output dimensions of 100 × 50 × 40 mm presented in Fig. 1b.

Finally, the multiplexer together with the T-DCF was assembled into the water-cooled amplifier housing with internal grooves enabling mechanical support and efficient cooling of an active tapered fiber (Fig. 1c).

### Seed source

**Figure 2:**
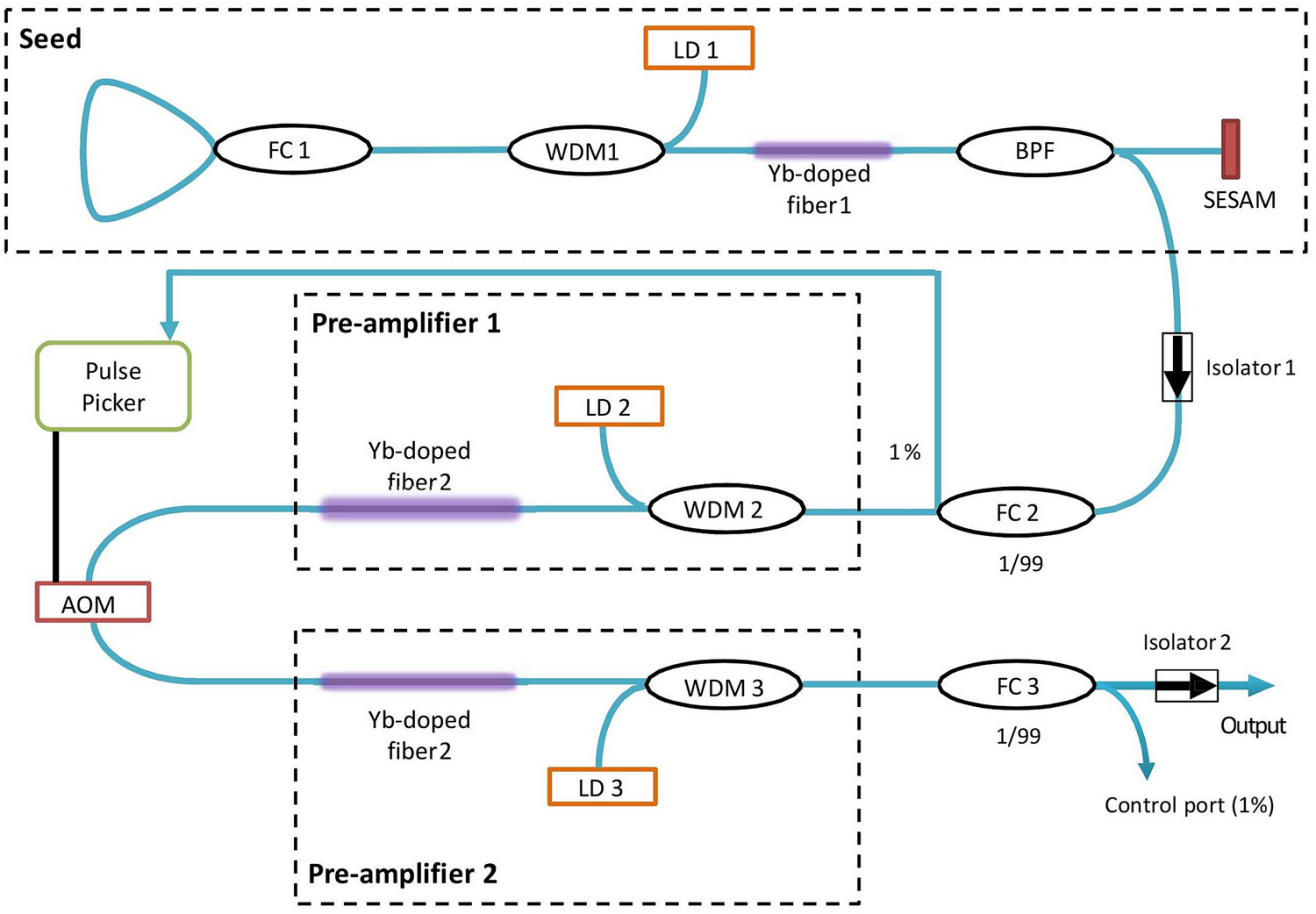
Schematic of the picosecond seed source with two pre-amplifiers (*FC* fiber coupler, *WDM* multiplexer, *BPF* Bandpass Filter/Tap Hybrid, *AOM* acousto-optic modulator, *LD* laser diode).

The polarization-maintaining seed source comprised four stages: a SESAM (semiconductor saturable absorber mirror) mode-locked fiber laser, an acousto-optical modulator (AOM-based pulse-picker), and two in-core pumped single-mode fiber pre-amplifier cascades (Fig. 2). The all-normal dispersion mode-locked fiber laser had a linear cavity design. It generated a pulse train with the repetition rate of 34 MHz and 41.5 ps pulse duration with average power up to 10 mW at 1040 nm wavelength. An Yb-doped fiber (Nufern PM980-XP) with 6/125 μm core-cladding diameters and 30 cm length was used as a laser gain medium and pumped by 976 nm laser diode via a wavelength-division multiplexer. A loop mirror was used as one of the cavity mirrors, and it was based on 50/50 fiber splitter with two output ports spliced together to form a loop. The second resonator mirror was a SESAM with 29% modulation depth, 1 ps relaxation time and working wavelength range of 980–1060 nm. The seed laser generated pulses with a full width at half maximum equaled to 0.9 nm at the central wavelength 1038 nm.

The pre-amplifiers 1 and 2 were implemented by using an Yb-doped fiber (an absorption equaled to 400 dB/m at 976 nm) with 6 µm and 10 µm core diameter, correspondingly, and numerical aperture (NA) equaled to 0.16. The preamplifiers were spliced together using the standard program for single-mode PM fibers of the Ericsson splicer. The splice loss as low as 0.02 dB was achieved. The pre-amplifiers were in-core pumped by 400 mW single-mode laser diodes. An AOM-based pulse picking system allows reduction of pulse repetition rate by selectively transmitting each i-th (i = 1, 2, 3, etc.) pulse from the SESAM mode-locked fiber laser towards power amplifier. The pulse picker electronics counts pulses and opens AOM only to transmit the pulse with user-specified number. This is the traditional technique for controlling repetition rate in mode-locked fiber lasers described in details, for example, in^14^. It is obvious that reduction of the pulse repetition rate leads to reduction of average output power, which should be compensated by increasing of the pump power in the second preamplifier. The repetition rate was varied from the fundamental value of 34 MHz down to 100 kHz. At the constant pump level and, accordingly, the output average power, a decrease in the pulse repetition rate leads to an increase in the energy and the peak power of the pulse. When using a laser for precision processing of materials, this supplements the flexibility in the operational regime expanding a range of the processed materials by one system.

**Figure 3:**
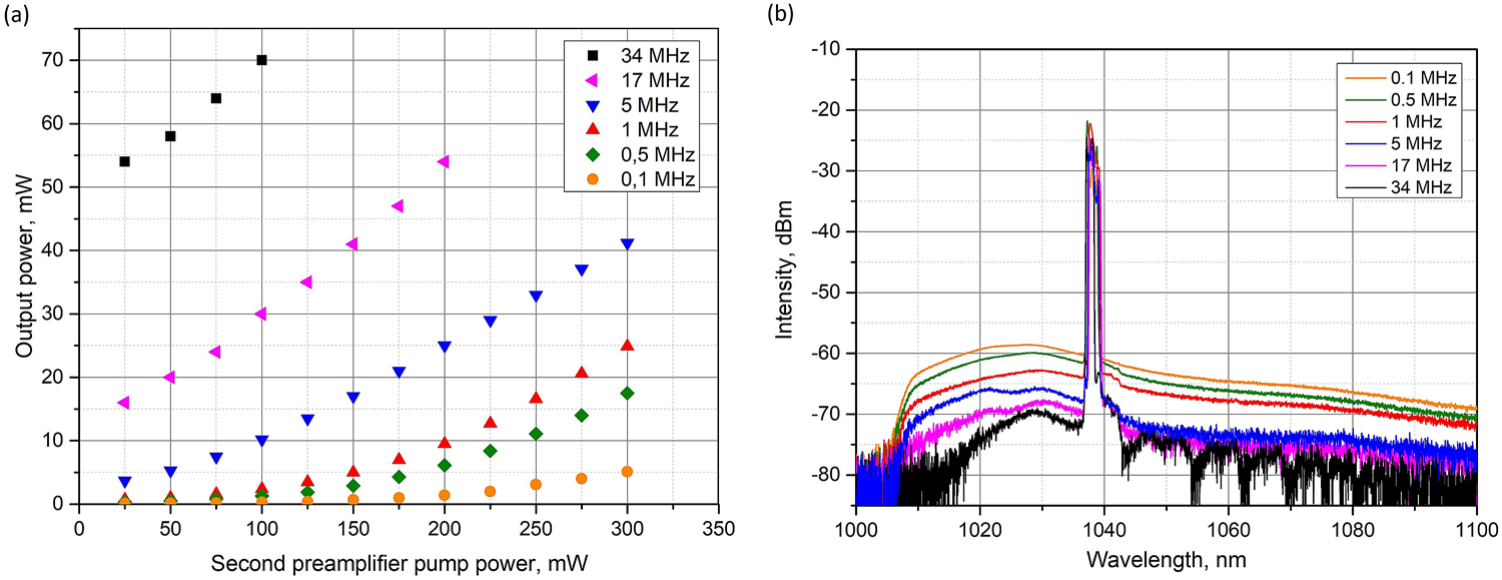
(**a**) Output power of the seed source versus the pump power from the second pre-amplifier pump diode at the various repetition rates. (**b**) The output spectra of the picosecond seed source after two pre-amplifiers at the different repetition frequencies.

The second pre-amplifier consisted of 10 µm fibers components, which enabled to obtain higher output power, as well as to reduce the probability of nonlinear effects (SRS) and to increase a peak power of the amplified signal. This pre-amplifier raised the output power to the sufficient level to feed the high-power tapered amplifier. The maximum output average power of the seed source was dependent on the repetition rate. The measured dependence is shown in Fig. 3a. The maximum output power was 70 mW at the fundamental repetition rate of 34 MHz and 5 mW at repetition rate of 100 kHz.

The output spectra of the seed source at different repetition rates are presented in Fig. 3b. The spectra corresponding to the different repetition rates were obtained at the same average output power equalled to 5 mW. The spectra were recorded using an optical spectrum analyzer Ando AQ6317B with 0.1 nm resolution bandwidth and output power was measured by Thorlabs PM100D power meter. The seed laser generated 41.5 ps pulses with a full width at half maximum equaled to 0.9 nm at the central wavelength 1038 nm.

One of the remarkable properties of T-DCF is possibility to efficiently amplify relatively small input signal^7^, therefore, even though the output power of the seed laser at 100 kHz repetition rate was at the level of 5 mW only, we still could get a reasonable contrast between ASE and signal power. The repetition rate was an integer number of the fundamental repetition rate according to the principle of the pulse-picker operation, and it was limited by 100 kHz due to low duty cycle of the laser at this frequency, and higher fraction of ASE raised in the amplification channel.

## Results and discussion

**Figure 4:**
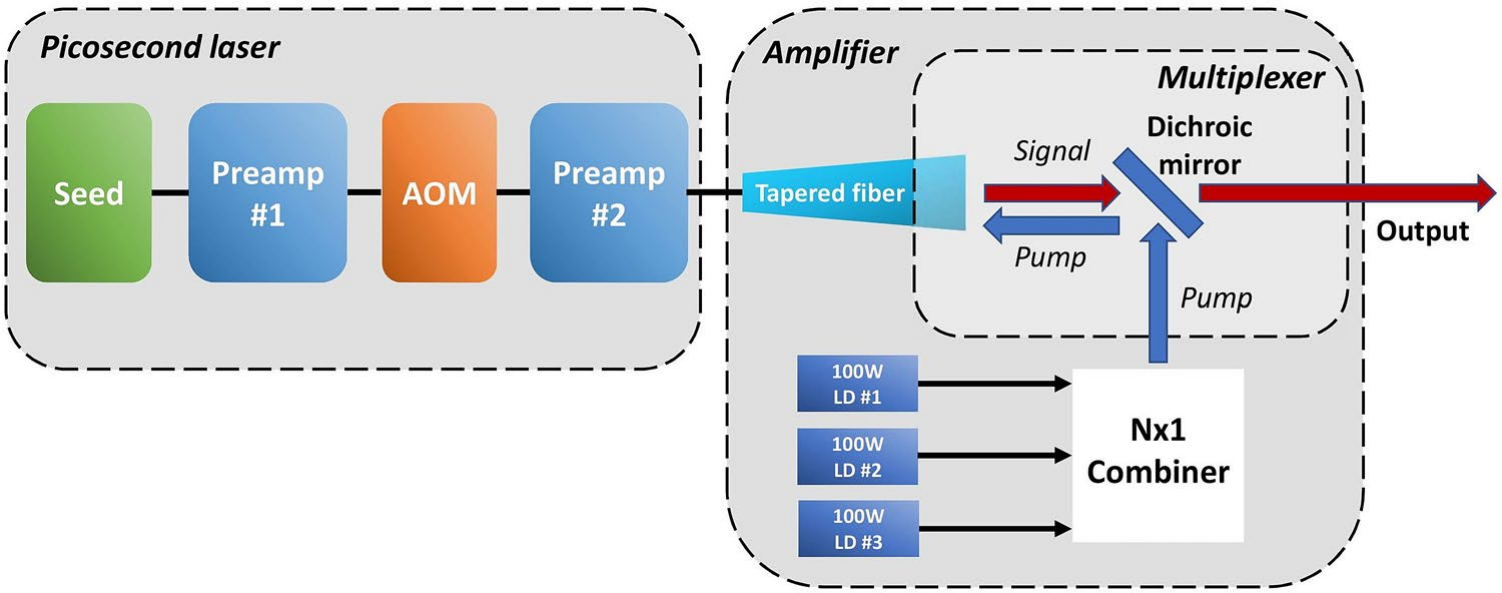
Schematic of the high-power picosecond laser consisting of a mode-locked fiber laser with two pre-amplifier stages and a boost amplifier based on an active tapered fiber.

The schematic of the complete integrated high-power picosecond MOPA system based on a picosecond seed source and an active tapered fiber amplifier is shown in Fig. 4. The tapered amplifier was pumped by three 100 W pump diodes via the packaged multiplexer described above.

The input signal of the high-power amplifier based on a tapered fiber should be carefully adjusted for the optimized performance. The feature of the tapered fiber amplifier (particularly due to its longitudinal geometry) that it requires small input signal level to increase the thresholds of the nonlinear effects. If the input power is too high, then in the narrow part of the tapered fiber it is amplified further resulted in Raman appearance. In contrast, when the input signal level is low, its amplification goes slowly to the similar level, whereas the fiber core increases significantly. This trick allows increasing the threshold of nonlinear effects. The input level of the signal was carefully adjusted to obtain the highest possible average power at the output. This procedure has been carried for each repetition rate separately.

**Figure 5:**
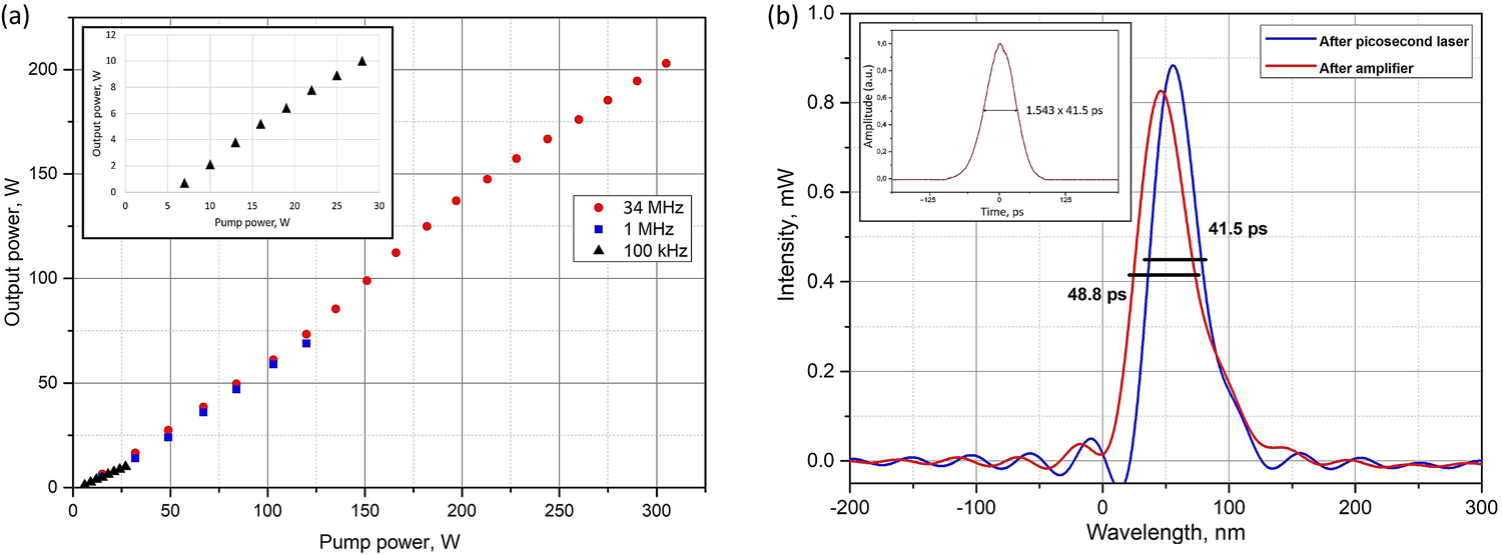
(**a**) Output power of the laser system versus total launched pump power at 34 MHz, 1 MHz and 0.1 MHz. Inset: the enlarged figure of the output power dependence at 0.1 MHz. (**b**) Pulse shape at the output of picosecond laser and the boost amplifier and autocorrelation trace of the seed source (sech^2^ pulse shape) (insert).

Figure 5a shows the average power at the laser system output as a function of the additive pump power at the fundamental frequency of 34 MHz, 1 MHz and 100 kHz. As it can be seen, the slope efficiency remained nearly the same (~ 71%) for different repetition rates. The pulse durations at the output of the seed source and the boost amplifier were measured by using 20 GHz oscilloscope Keysight UXR0204A with 33 GHz high sensitivity optical-to-electrical converter Keysight N7004A. The pulse duration before amplifier (repetition rate was 34 MHz, signal power was 54 mW) was 41.5 ps, and after amplifier (same signal parameters and output power 50 W) was 48.8 ps (Fig. 5b). The pulse duration increases during amplification, which is in line with previously observed experimental results^15^. The temporal pulse profile measured by an autocorrelator Avesta IRA-VISIR is shown in insert of Fig. 5b confirming the pulse duration measured by the oscilloscope.

**Figure 6:**
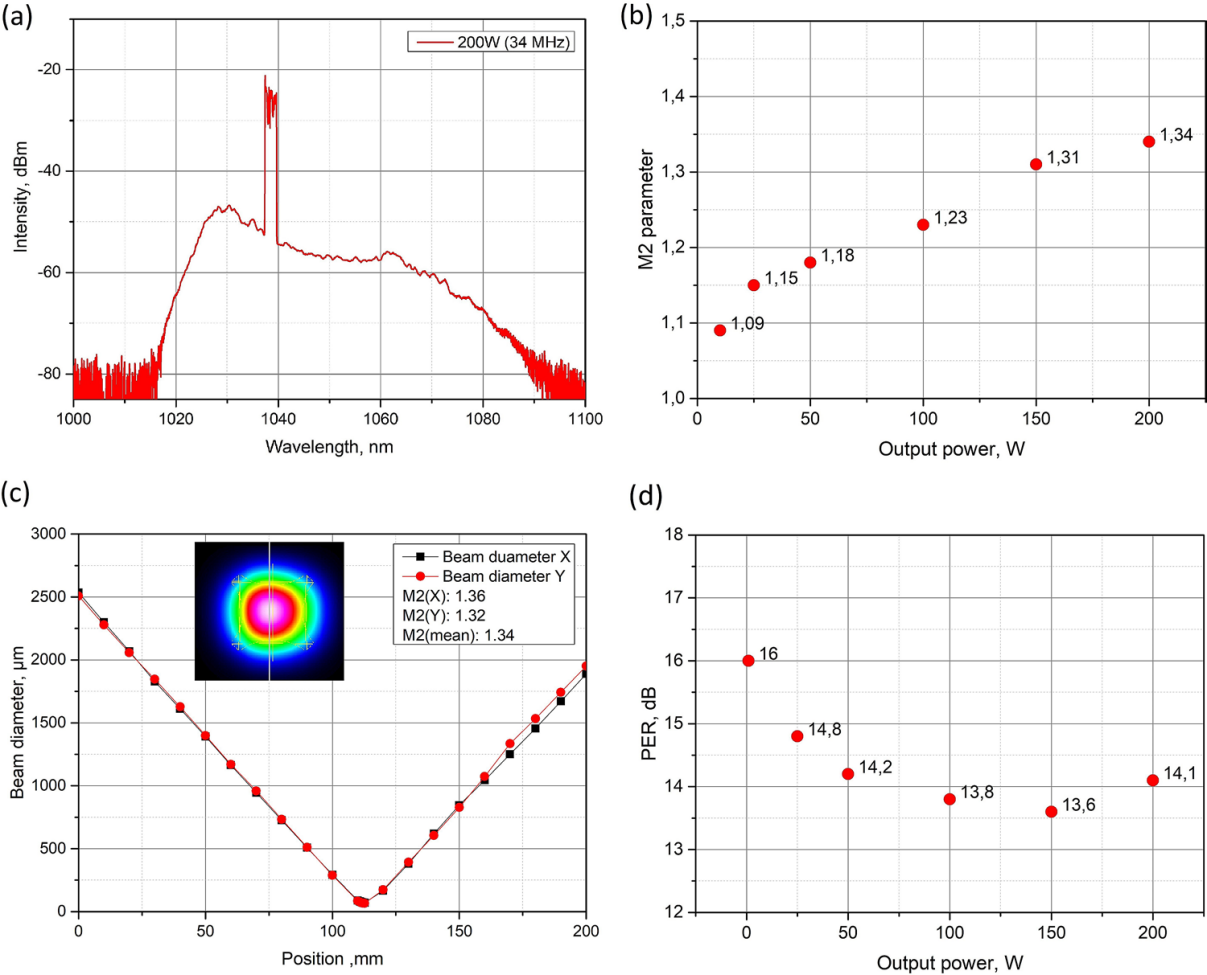
(**a**) The spectrum at the output of the T-DCF amplifier at 34 MHz and 200 W of average power. (**b**) M^2^ parameter versus output power. (**c**) The result of the measured M^2^ at 200 W output power and the far field beam profile. (**d**) Polarization extinction ratio (PER) versus output power of the T-DCF amplifier.

The maximum average power achieved at the system output was 200 W at 34 MHz repetition rate. The signal power at the input of the tapered amplifier was 54 mW for the best performance, and the total pump power was 300 W. The signal gain was equaled to 35.7 dB. The pulse energy at the fundamental frequency was 5.9 µJ and the peak power was 0.11 MW, which is already sufficient for high speed machining^16^. We would like to note that the pulse energy was calculated by extracting of the ASE (amplified spontaneous emission) part. The spectrum of the output radiation at the maximum output power of 200 W at 34 MHz repetition rate is shown in Fig. 6a. In this case, the system performance has been limited by the pump power and the SRS threshold has been achieved.

We investigated the beam quality factor M^2^ at the system output within the whole range of the output power for 34 MHz repetition rate. The M^2^ parameter was measured by a Thorlabs M2MS-BC106VIS measurement system, and the results are presented in Fig. 6b. The beam quality did not reveal significant degradation with the increase of the output power. The M^2^ parameter was equal to 1.34 at the maximum output power of 200 W (Fig. 6b). Figure 6c shows the far field beam profile taken with the same camera at 200 W output power. As it can be seen the fundamental mode is slightly constrained from one side resulting in small deviation of the mode shape from circular. However, we would like to note that monitoring of the output power at the maximum level has not revealed any power drift or instability; therefore, we can conclude the presence of one mode only even at the maximum power.

We have also performed the investigation of polarization extinction ratio (PER) evolution as a function of the output radiation (Fig. 6d). The PER slightly decreased with the increase of the output power approaching minimum value of 13.6 dB at 150 W power. We assume that increase of PER at 200 W is due to measurement inaccuracy at high average power.

**Figure 7:**
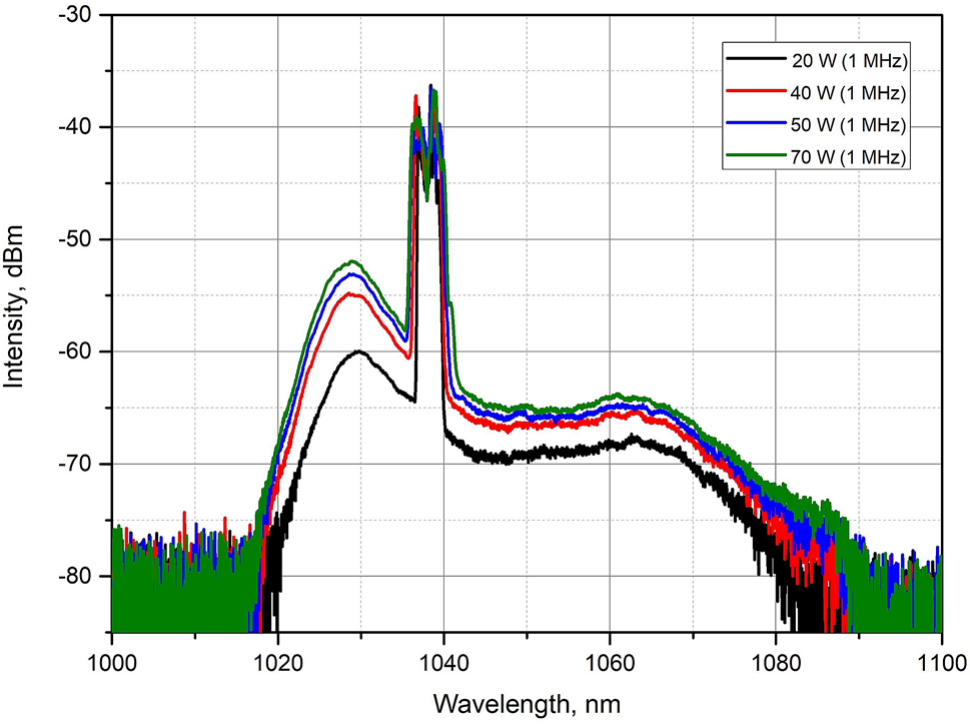
Spectra after the T-DCF amplifier at 1 MHz at the average output power 20 W, 40 W, 50 W and 70 W.

Next, the dependence of output spectra on the pump power was studied at 1 MHz pulse repetition rate. The input signal power was 17 mW at 1040 nm. The pump power of 120 W allowed obtaining of 70 W of the average output power with the total signal gain value equaled to 36.2 dB. Figure 7 shows the output spectra for four different average power levels. As can be seen from Fig. 7, a signal amplification leads to a broadening of its spectrum^17^. To calculate pulse energy correctly, we have determined percentage of spectral power in the signal band. At 70 W output power 86% of the power was in the signal band resulting in the pulse energy of 60 µJ and corresponding peak power of 1.17 MW without presence of Raman peaks in the signal spectrum.

We would like to note that with the decrease of the repetition rate, the time between neighbouring pulses increases resulting in stronger amplification of the ASE under continuous pumping. As consequence, the SNR decreases and further pumping leads to the worse SNR; therefore, we performed the results with SNR exceeded 15 dB only.

**Figure 8:**
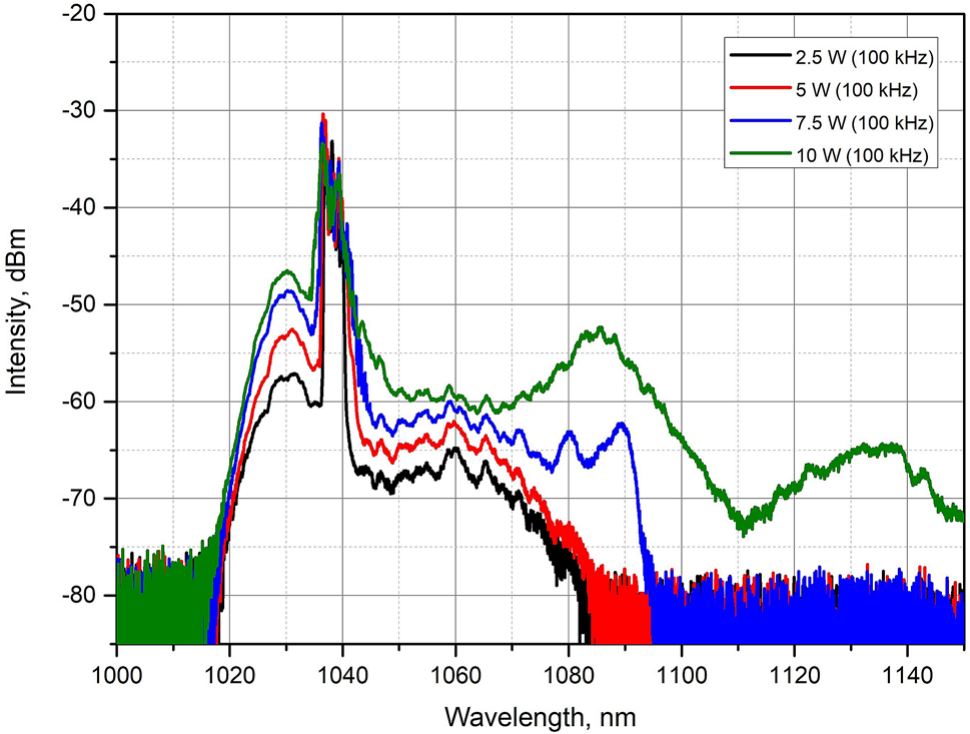
Spectra after the T-DCF amplifier at 100 kHz with varied pump level.

Next, we reduced the pulse repetition rate down to 100 kHz, which allowed us to increase the peak power further while reducing the average power to stay within nonlinearities-free operation. The input signal power, in this case, was 5 mW. At the maximum pump power of 27 W, we obtained 10 W of the average power. However, in this case, the SRS signal was clearly visible on the spectrum (Fig. 8). It was possible to minimize the SRS peak by decreasing the pump level down to 21 W, which gave 7.5 W of the average power. The pulse energy was determined following the same procedure as for 1 and 34 MHz resulting in 65 µJ in this case. Correspondingly, the peak power of 1.26 MW was obtained with the signal gain equaled to 31.8 dB.

The comprehensive experimental investigation of the system revealed that a T-DCF-based amplifier was a robust instrument for power scaling of ultra-short pulses due to a unique longitudinal geometrical profile. It can be considered as a continuous chain of amplifiers with a smooth increase of a fiber diameter from typical single-mode to large-mode-area values. Consequently, it tolerates a few milliwatts input signal, and capable to amplify it further up to 200 W average power. Therefore, it allows simplification of the system by reduction of single-mode pre-amplifiers stages. Due to the same variation of the fiber diameter along its length, the threshold of nonlinear effects is elevated enabling to achieve high peak power (1.26 MW) and pulse energy (65 µJ) at the repetition rate as low as 100 kHz with reasonable SNR level (> 15 dB). Due to smooth transition from single-mode fiber’s input to multimode output resulting in mode content preservation, the T-DCF-based amplifier delivers a near-diffraction-limited beam (M2 = 1.3) within the whole power range.

## Conclusion

In this paper, we demonstrated an efficient and compact all-fiber polarization-maintaining high-power picosecond laser system based on an active tapered double clad fiber. It was possible to implement compact amplifier design thanks to the development of a small size micro-optics-based pump multiplexer capable to deliver up to 300 W of pump power to T-DCF aperture. We have reached 65 µJ level of the pulse energy with peak power more than 1.2 MW at 100 kHz repetition rate while maintaining near diffraction-limited beam quality. The maximum average power obtained in the system was 200 W at 34 MHz repetition rate and 41.5 ps pulse duration with corresponding M^2^ ~ 1.34. To the best of our knowledge, these are the record values obtained for the polarization-maintaining fiber amplifiers by the direct amplification of a picosecond signal without involvement of the CPA technology^18^. This combination of the high peak power and high average power is a unique property of amplifiers based on the T-DCF. This opens the door for practical application of the T-DCF-based all-fiber picosecond lasers for industrial high precision machining.
